# Supplementary figures and images for: Changes in inflammatory and vasoactive mediator profiles during valvular surgery with or without infective endocarditis: A case control pilot study
Source: PLoS One. 2020 Feb 3;15(2):e0228286. doi: 10.1371/journal.pone.0228286 (PMC6996967; doi:10.1371/journal.pone.0228286)

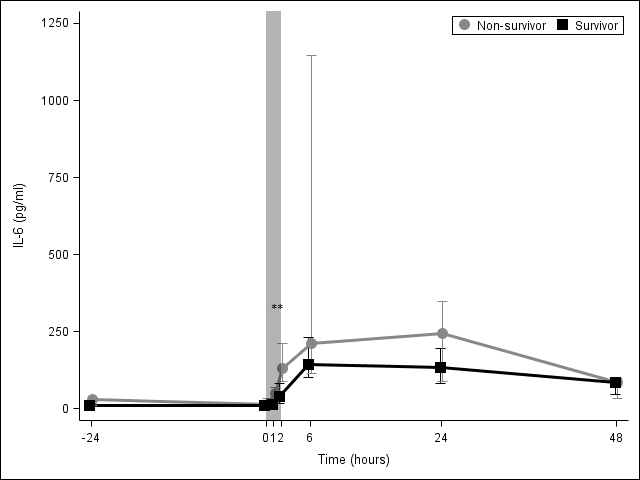

Supplement: S1 Fig — This is the S1 Fig legend: Bright line: non-survivors; dark line survivors; 0 on the x-axis represents the beginning of cardiopulmonary bypass (CPB); the shaded area represents the CPB time; IL: interleukin; *: p<0.05. (TIF) [file pone.0228286.s002.tif]

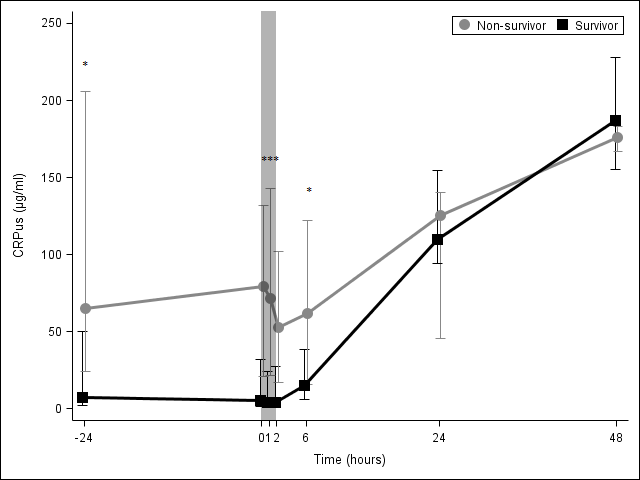

Supplement: S2 Fig — Bright line: non-survivors; dark line survivors; 0 on the x-axis represents the beginning of cardiopulmonary bypass (CPB); the shaded area represents the CPB time; CRP: C—reactive protein; *: p<0.05. (TIF) [file pone.0228286.s003.tif]

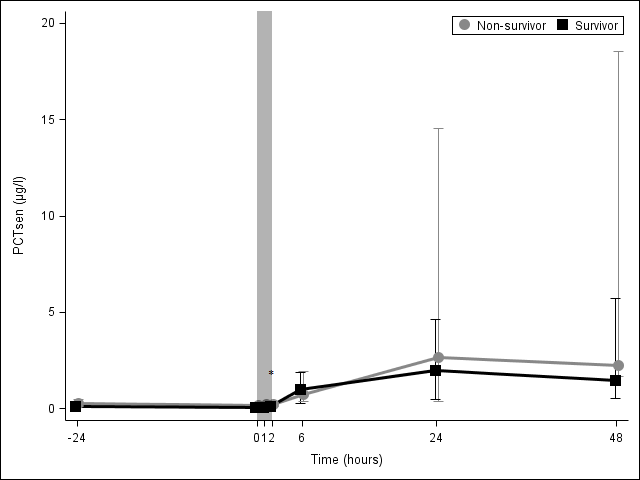

Supplement: S3 Fig — Bright line: non-survivors; dark line survivors; 0 on the x-axis represents the beginning of cardiopulmonary bypass (CPB); the shaded area represents the CPB time; PCT: procalcitonin; *: p<0.05. (TIF) [file pone.0228286.s004.tif]

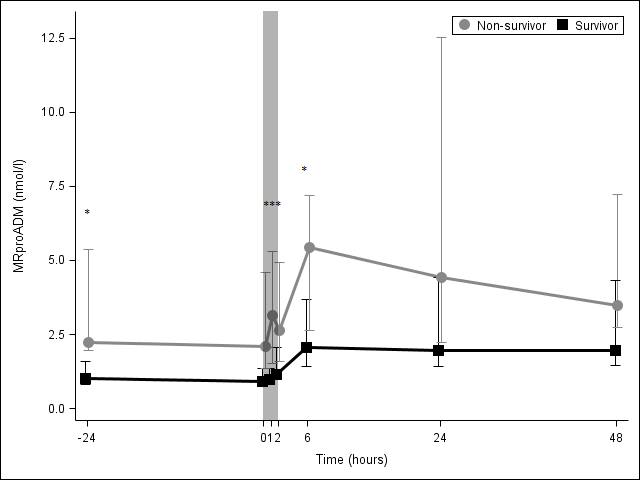

Supplement: S4 Fig — Bright line: non-survivors; dark line survivors; 0 on the x-axis represents the beginning of cardiopulmonary bypass (CPB); the shaded area represents the CPB time; MR-proADM: midregional pro adrenomedullin; *: p<0.05. (TIF) [file pone.0228286.s005.tif]

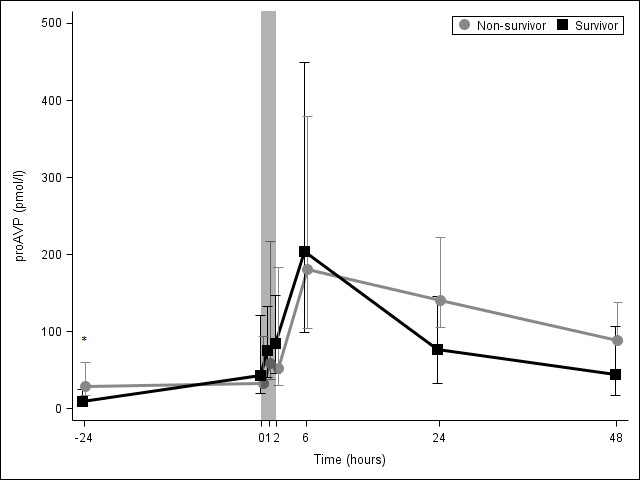

Supplement: S5 Fig — Bright line: non-survivors; dark line survivors; 0 on the x-axis represents the beginning of cardiopulmonary bypass (CPB); the shaded area represents the CPB time; proAVP: copeptin pro vasopressin; *: p<0.05. (TIF) [file pone.0228286.s006.tif]

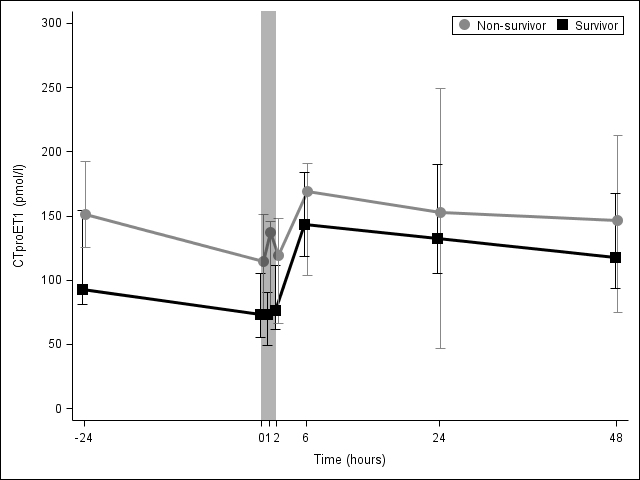

Supplement: S6 Fig — Bright line: non-survivors; dark line survivors; 0 on the x-axis represents the beginning of cardiopulmonary bypass (CPB); the shaded area represents the CPB time; CT-proET-1: C-terminal pro endothelin-1. (TIF) [file pone.0228286.s007.tif]

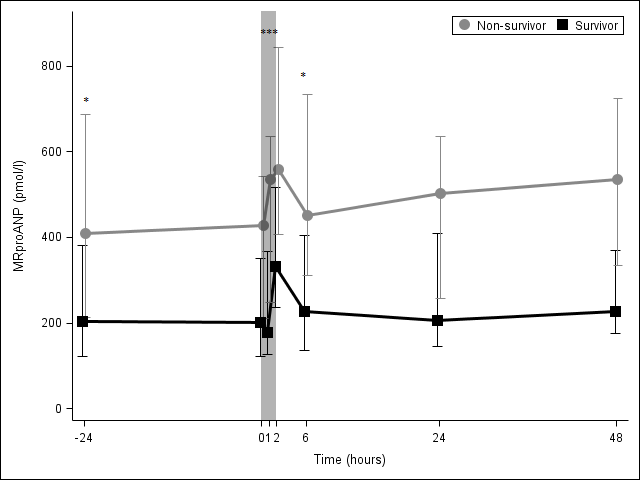

Supplement: S7 Fig — Bright line: non-survivors; dark line survivors;0 on the x-axis represents the beginning of cardiopulmonary bypass (CPB); the shaded area represents the CPB time; MR-proANP: midregional pro atrial natriuretic peptide; *: p<0.05. (TIF) [file pone.0228286.s008.tif]

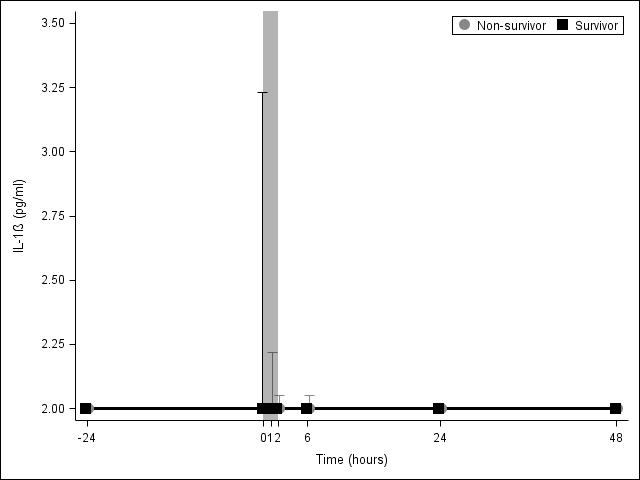

Supplement: S8 Fig — Bright line: non-survivors; dark line survivors; 0 on the x-axis represents the beginning of cardiopulmonary bypass (CPB); the shaded area represents the CPB time; IL: interleukin. (TIF) [file pone.0228286.s009.tif]

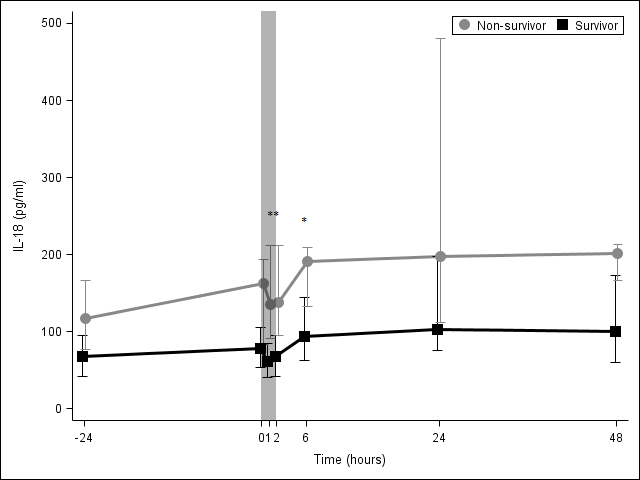

Supplement: S9 Fig — Bright line: non-survivors; dark line survivors; 0 on the x-axis represents the beginning of cardiopulmonary bypass (CPB); the shaded area represents the CPB time; IL: interleukin;*: p<0.05. (TIF) [file pone.0228286.s010.tif]

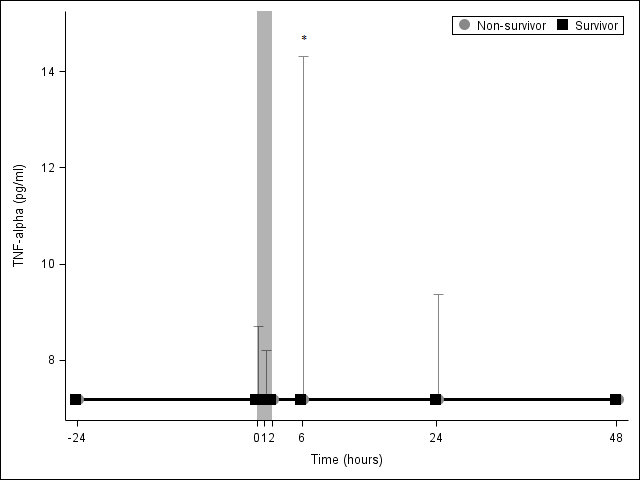

Supplement: S10 Fig — Bright line: non-survivors; dark line survivors;0 on the x-axis represents the beginning of cardiopulmonary bypass (CPB); the shaded area represents the CPB time; TNF: tumour necrosis factor;*: p<0.05. (TIF) [file pone.0228286.s011.tif]

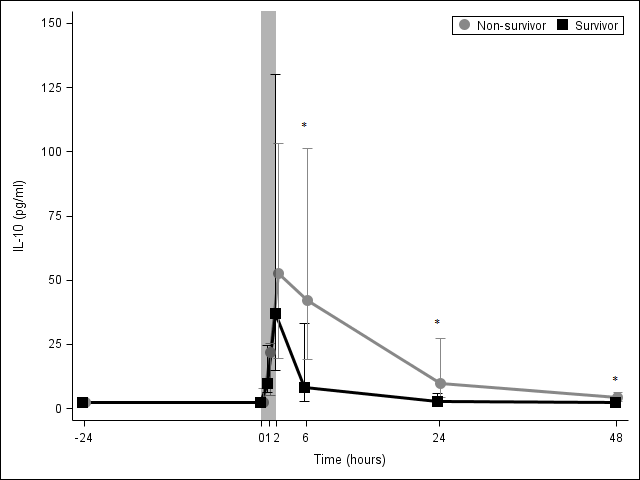

Supplement: S11 Fig — Bright line: non-survivors; dark line survivors; 0 on the x-axis represents the beginning of cardiopulmonary bypass (CPB); the shaded area represents the CPB time; IL: interleukin;*: p<0.05. (TIF) [file pone.0228286.s012.tif]
