# Supplementary material for: Changes in inflammatory and vasoactive mediator profiles during valvular surgery with or without infective endocarditis: A case control pilot study
Source: PLoS One. 2020 Feb 3;15(2):e0228286. doi: 10.1371/journal.pone.0228286 (PMC6996967; doi:10.1371/journal.pone.0228286)
Supplement: S1 File — (DOCX) [file pone.0228286.s013.docx]

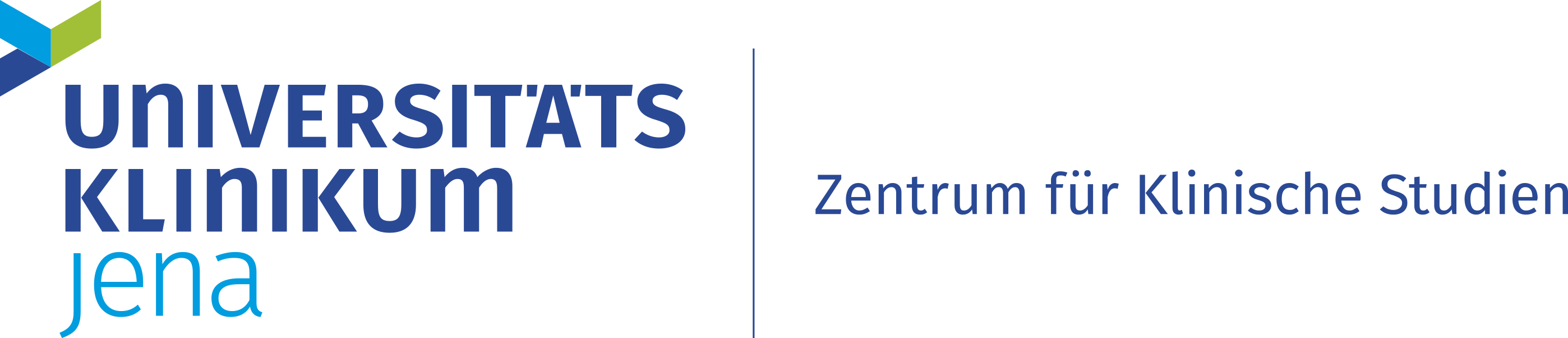


Study Protocol

***Inflammatory and vasoactive mediator profiles***

***and pathogen characterization***

***during heart valve replacement surgery***

**REMOVE-Pilot**

Principal Investigator:

**Mahmoud Diab, MD**

Department of Cardiac and Thoracic Surgery

University Hospital Jena

Erlanger Allee 101

07747 Jena

Partially supported by B.R.A.H.M.S GmbH, part of Thermo Fisher Scientific

Version Final

**Table of contents**

[1. Signature page 3](#_Toc421627562)

[2. List of abbreviations 4](#_Toc421627563)

[3. Responsible parties 5](#_Toc421627564)

[4. Abstract 8](#_Toc421627565)

[5. Amendments and updates 10](#_Toc421627566)

[6. Milestones 10](#_Toc421627567)

[7. Rationale and background 11](#_Toc421627568)

[8. Research questions and objectives 11](#_Toc421627569)

[8.1. Auxiliary objectives requiring external cooperation 12](#_Toc421627570)

[9. Research methods 13](#_Toc421627571)

[9.1. Study design 13](#_Toc421627572)

[9.2. Setting 13](#_Toc421627573)

[9.3. Variables 15](#_Toc421627574)

[9.4. Data sources 15](#_Toc421627575)

[9.5. Study size 16](#_Toc421627576)

[9.6. Data management 16](#_Toc421627577)

[9.7. Data analysis 17](#_Toc421627578)

[9.8. Quality control 18](#_Toc421627579)

[9.9. Limitations of the research methods 18](#_Toc421627580)

[10. Protection of human subjects 19](#_Toc421627581)

[11. Management and reporting of adverse events/adverse reactions 19](#_Toc421627582)

[12. Plans for disseminating and communicating study results 20](#_Toc421627583)

[13. References 21](#_Toc421627584)

# Signature page

The signatories confirm herewith their approval of the present Study Protocol.

| ___________________________________ |  | ________________ |  | ________________ |
| --- | --- | --- | --- | --- |
| **Name: Mahmoud Diab, MD Principal Investigator** |  | **Date** |  | **Signature** |
| _________________________________ |  | _______________ |  | _______________ |
| **Name: Thomas Lehmann, PhD Biometrician** |  | **Date** |  | **Signature** |

# List of abbreviations

| AUC | Area under the curve |
| --- | --- |
| CFR | Code of federal regulations |
| CPB | Cardiopulmonary bypass |
| CRP | C-reactive protein |
| DNA | Deoxyribonucleic acid |
| EDTA | Ethylene diamine tetraacetic acid |
| eCRF | Electronic case report form |
| GCP | Good clinical practice |
| GLP | Good laboratory practice |
| ICU | Intensive care unit |
| IE | Infective endocarditis |
| IFN-γ | Interferon gamma |
| IL-1β | Interleukin 1beta |
| IL-6 | Interleukin 6 |
| IL-10 | Interleukin 10 |
| IL-18 | Interleukin 18 |
| IRB | Institutional review board |
| MS | Mass spectrometry |
| NGS | Next generation sequencing |
| PCT | Procalcitonin |
| Pro-ADM | Pro-Adrenomedullin |
| Pro-ANP | Pro-Atrial natriuretic peptide |
| Pro-AVP | Pro-Arginine-Vasopressin; Copeptin |
| mRNA | Messenger ribonucleic acid |
| SOFA | Sequential Organ Failure Assessment |
| SOP | Standard operating procedure |
| TNFα | Tumor necrosis factor alpha |
| VHD | Valvular heart disease |

# Responsible parties

| Principal investigator | Mahmoud Diab, MD  Department of Cardiac and Thoracic Surgery  University Hospital Jena  Erlanger Allee 101, 07747 Jena  Tel. 03641 9322978  E-Mail: [*mahmoud.diab@med.uni-jena.de*](mailto:mahmoud.diab@med.uni-jena.de) |
| --- | --- |
| Co-investigators | Prof. Torsten Doenst, MD  Department of Cardiac and Thoracic Surgery  University Hospital Jena  Erlanger Allee 101, 07747 Jena  Tel. 03641 9322901  E-Mail: *Doenst@med.uni-jena.de*  Christoph Sponholz, MD  Department of Anaesthesiology and Intensive Care Medicine  University Hospital Jena  Erlanger Allee 101, 07747 Jena  Tel. 03641 9322225  E-Mail: [*christoph.sponholz@med.uni-jena.de*](mailto:christoph.sponholz@med.uni-jena.de) |
| Scientific advisors | Prof. Frank M. Brunkhorst, MD  Jena University Hospital  Center for Clinical Studies  Salvador-Allende-Platz 27, 07747 Jena  Tel.: 03641-9323381  E-Mail: [*frank.brunkhorst@med.uni-jena.de*](mailto:frank.brunkhorst@med.uni-jena.de)  Prof. Michael Bauer, MD  Center for Sepsis Control and Care (CSCC)  University Hospital Jena  Erlanger Allee 101, 07747 Jena  Tel: 03641- 9323123  E-Mail: [*michael.bauer@med.uni-jena.de*](mailto:michael.bauer@med.uni-jena.de) |
| Responsible laboratories | Kristin Hagenow  Thermo Fisher Scientific Clinical Diagnostics B·R·A·H·M·S GmbH Neuendorfstrasse 25, 16761 Hennigsdorf Tel. 03302 883 661 E-Mail: [*kristin.hagenow@thermofisher.com*](mailto:kristin.hagenow@thermofisher.com)  Kai Sohn, PhD  Fraunhofer Institute for Interfacial Engineering and Biotechnology  Molecular Biotechnology/Functional Genomics  Nobelstrasse 12, 70569 Stuttgart  Tel. 0711 970 4055  E-Mail: [*kai.sohn@igb.fraunhofer.de*](mailto:kai.sohn@igb.fraunhofer.de) |

| Project Management | Vladimir Patchev, MD  Jena University Hospital  Center for Clinical Studies  Salvador-Allende-Platz 27, 07747 Jena  Tel.: 03641-9396650  E-Mail: *vladimir.patchev@med.uni-jena.de* |
| --- | --- |
| Biometrics | Thomas Lehmann, PhD  Jena University Hospital  Center for Clinical Studies  Salvador-Allende-Platz 27, 07747 Jena  Tel.: 03641-933610  E-Mail: *thomas.lehmann@med.uni-jena.de* |
| Data Management | Cornelia Eichhorn  Jena University Hospital  Center for Clinical Studies  Salvador-Allende-Platz 27, 07747 Jena  Tel.: 03641- 9396653  E-Mail: *cornelia.eichhorn@med.uni-jena.de* |

# Abstract

| **Title** | Inflammatory and vasoactive mediator profiles and pathogen characterization during heart valve replacement surgery |
| --- | --- |
| **Short title** | Remove-Pilot |
| **Rationale and background** | Infective endocarditis (IE) is a grave infectious disease with an incidence of  3-5 per 100 000 in the general population and exceeding 15 per 100 000 in persons aged above 65. The predominant causal pathogens are *Staphylococcus spp*. and *Enterococcus spp*., with increasing role of health care-associated infections. More than 50 % of IE patients undergo cardiac surgery which is associated with high mortality (6-25 % in the average and exceeding 40 % in certain patient cohorts).  Valvular heart disease (VHD) is another major indication for heart surgery. Its prevalence amounts to 2.5 %, with further increase to 8.5 % beyond the age of 65. Post-surgery mortality rates vary between 2.9 and 6.5 %.  Exaggerated release of inflammatory mediators and endogenous vasoactive substances resulting from the coincident infection and surgical stress plays a role in post-operative organ failure and altered immune defense, thus contributing to unfavorable post-operative outcome.  Cardiopulmonary bypass (CPB) itself, even in the absence of IE, has been shown to modify cytokine and vasoactive mediator release and may cause organ failure. Tracing of release profiles of inflammatory cytokines and vasoactive mediators and their correlation with postoperative organ dysfunction in cardiac surgery for IE or non-IE patients aims at the assessment of the prognostic validity of these biomarkers and the evaluation of measures for their pro-active clearance during the surgical intervention.  Induction of inflammatory mediators and their temporal release profile may vary depending on the involved pathogens, which cannot be always identified by conventional techniques (blood culture). Since it is conceivable that identification of the involved pathogen could explain differences in cytokine secretory patterns in IE, use of advanced molecular technologies (NGS) will support the clarification of such relations. Analysis of transcripts encoding inflammatory and vasoactive mediators in blood cells will enable the surveillance of temporal oscillations in their profiles during the observation time frame. Transcriptome analysis of identified putative pathogens can also disclose features of antibiotic resistance. |
| **Research question and objectives** | - Release profiles of selected inflammatory and vasoactive mediators in the course of, and over 48 hours after cardiac surgery with CPB for infective endocarditis and valvular heart disease - Identification of involved pathogens by NGS of cell-free circulating nucleic acids - Temporal profiles of transcripts encoding inflammatory and vasoactive mediators in patients undergoing cardiac surgery with CPB - Identification of pathogen-specific resistance-coding transcripts in whole blood RNA extracts |
| **Design** | Monocentric case-control diagnostic study |
| **Population** | Patients diagnosed with infective endocarditis or valvular heart disease, undergoing cardiac surgery with cardiopulmonary bypass |
| **Endpoints** | - Plasma profiles of inflammatory biomarkers at defined time points in the course of the surgical intervention (baseline, before introduction of CPB, 1 hour after introduction of CPB, disconnection of CPB, 6, 24 and 48 hours post-surgery)   - Procalcitonin   - C-reactive protein   - Endothelin 1   - TNFα   - IL-1β   - IL-6   - IL-10   - IL-18 - Plasma profiles of inflammation-related vasoactive mediators at defined time points in the course of the surgical intervention (baseline, before introduction of CPB, 1 hour after introduction of CPB, disconnection of CPB, 24 and 48 hours post-surgery)   - pro-Adrenomedullin (pADM)   - pro-Arginine Vasopressin (pAVP)   - pro-Atrial Natriuretic Peptide (pANP) - Changes in organ dysfunction 24 and 48 h post-surgery, disclosed by  Δ SOFA score as compared to pre-surgery status - Use and duration of renal replacement therapy - Cumulative doses of concomitant medications (vasopressors, corticoids, prostaglandins) applied during the surgery and over 48 h thereafter - In-hospital mortality over 30 days post-surgery - Microbial DNA profiles in blood plasma at selected time points and in valve tissue samples - Abundance of mRNA encoding inflammatory and vasoactive mediators in whole blood at selected time points - Abundance of bacterial mRNA encoding antibiotic resistance factors in whole blood - **In cooperation with Thermo Fisher Scientific (see 8.1):** Plasma profiles of inflammation-related protein fragmentation products denoted by MS-based fragmentation signatures CDB-04, 19, 26, 28, 31, 34, 50, 59, 66, 67 |
| **Inclusion criteria** | - Patients with infective endocarditis in accordance with Duke criteria undergoing cardiac surgery  - Patients undergoing cardiac valvular surgery in absence of IE  - Age > 18 years  - Signed informed consent |

| **Exclusion criteria** | - Glucocorticoid dosage above Cushing threshold (Prednisolone 7.5 mg/d or  Cortisol 30 mg/d)  - Severe neutropenia (below 1000/mm^3^)  - Immunosuppression  - Pregnancy | |
| --- | --- | --- |
| **Projected patient number** | - totally 120 participants, comprising two groups of 60 patients per study arm (IE and VHD, respectively)  - interim data analysis after recruitment of “batches” of 20 patients per arm  - optional recruitment stop, as soon as interim analysis reveals significant between-arms differences in biomarker profiles | |
| **Centers** | Department of Cardiac and Thoracic Surgery, University Hospital Jena | |
| **Timetable** | Recruitment duration | 24 months |
|  | First patient in | 01 April 2016 |
|  | Last patient out | 31 March 2018 |
|  | Duration in individual patients | 30 days |

#

# Amendments and updates

| **Number** | **Date** | **Section of study protocol** | **Amendment or update** | **Reason** |
| --- | --- | --- | --- | --- |
|  |  |  |  |  |
|  |  |  |  |  |
|  |  |  |  |  |

# Milestones

| **Milestone** | **Planned date** |
| --- | --- |
| Data base closure | One month after last patient out |
| End of statistic evaluation | Two months after data base closure |
| Final report | Three months after completion of statistic processing |

# Rationale and background

Infective endocarditis (IE) affects 1-10/100,000 persons per year and is associated with up to 30% hospital mortality ***[1-3]***. Surgical treatment is necessary in about 50% of patients and is associated with in-hospital mortality as high as 15-25% and 1-year mortality of 40% ***[1, 4].*** The postoperative course of patients with IE is often complicated with a varying degree of circulatory failure i.e. hypotension, decreased systemic vascular resistance, despite high cardiac output, adequate fluid resuscitation, and adrenergic vasopressor administration in up to 10-28% of cases ***[5-7]***. If septic shock complicates the course of the disease the mortality can reach up to 75-100 % in some studies ***[7, 8]***. Development of septic shock in patients with IE has been associated with dramatic deterioration of the prognosis and several-fold mortality increase ***[9]***. In our center, septic shock leading to multiple organ failure was the main cause of death in 60% of patients who died after surgical procedures for IE ***[10]***.

The mechanisms that trigger septic shock in endocarditis patients are also not fully understood and warrant further investigation ***[9]***. Increased release of pro-inflammatory mediators (IL-6, IL-8, and IFN-γ) has been associated with unfavorable outcome in severe sepsis and severe respiratory illness ***[12-14]***. On the other hand, available information on the role of these cytokines in IE is scarce. Elevated IL-6, IL-8 and IFN-γ plasma levels are reportedly associated with an unfavorable outcome in patients with prosthetic-valve IE ***[15]***. Furthermore, the local occurrence of IL-8-, but not TNFα-containing cells in valve tissue of IE patients has been considered as a potential marker of disease activity ***[16]***.

# Research questions and objectives

IE is a grave inflammatory condition that is associated with substantial activation of the innate immune system, including overproduction and uncontrolled release of inflammatory mediators. There is a compelling evidence that a continuous “inflammatory cytokine storm” can initiate cytotoxic effects in several organs ***[***[***1***](#_ENREF_1)***7]*** and cause “immune paralysis” ***[***[***1***](#_ENREF_2)***8]*** resulting in organ failure and/or increased susceptibility to superimposed infection. It is, thus, conceivable that increased release of inflammatory mediators may significantly contribute to short-term morbidity and mortality in IE.

Cardiovascular surgery using extracorporeal circulation causes a systemic inflammatory response which often results in severe organ dysfunction and increased postoperative mortality ***[19]***. Comparison of the release profiles of cytokines involved in the response to cardiopulmonary bypass (CPB) in patients undergoing surgery for IE and VHD may provide valuable knowledge and suggest approaches resulting in outcome improvement. Thus, clarification of the fractional contribution of infectious background, surgical trauma and CPB to the perioperative “spill-over” of inflammatory and vasoactive mediators might bring about measures aiming at perioperative reduction of the exposure to these substances and, thereby, improve survival of endocarditis patients undergoing cardiac surgery. In view of the superimposition of challenges capable of inducing inflammatory mediators, the examination should involve a comprehensive array of established inflammation biomarkers (CRP, PCT, Endothelin, TNFα, IL-1β, IL-6, IL-10, and IL-18).

Another possible determinant of cytokine release profiles is the nature of the involved pathogens ***[20, 21]***. However, conventional blood culture examinations fail to disclose the presence of a putative pathogen in a sizeable fraction of patients with IE ***[22]***, thus requiring the use of advanced molecular technologies ***[23]*** in the pursuit of this issue.

Scrutiny of involved pathogens by Next Generation Sequencing of cell-free circulating nucleic acids (besides data obtained from routine blood cultures), identification of pathogen-specific resistance-coding transcripts in whole blood RNA extracts and comparison of the temporal profiles of transcripts encoding inflammatory and vasoactive mediators and their circulating levels are expected to provide valuable non-conventional options for the adjustment of the therapeutic strategy and prognostic assessment.

The necessary technology and qualified personnel for the investigation of these parameters can be provided by the Fraunhofer Institute of Interfacial Engineering and Biotechnology.

The mechanism of circulatory failure complicating the postoperative course of endocarditis has not been completely elucidated. Some presumed mechanisms include a combination of endothelial injury, arginine-vasopressin system dysfunction, and release of vasodilatatory mediators ***[11]***. It is, thus, conceivable to monitor the intra- and postoperative release profiles of vasoactive mediators that affect cardiovascular function and are regulated by both, infectious ***[24-26]*** and general or cardiogenic stressful stimuli (pro-Adrenomedullin, pro-Vasopressin, pro-Atrial natriuretic peptide) ***[27, 28-31]***.

## Auxiliary objectives requiring external cooperation

Further useful information can be extracted from the examination of dynamic changes in plasma profiles of inflammation-related protein fragmentation products of putative, though insufficiently characterized relevance, denoted by MS-based fragmentation signatures CDB-04, 19, 26, 28, 31, 34, 50, 59, 66, 67. At present, the examination of the parameters listed above can be accomplished solely by technologies and know-how available at B.R.A.H.M.S. GmbH (Thermo Fisher).

# Research methods

## Study design

The study is conceived as a case-control observation and will be carried out in a single center.

## Setting

Subject to recruitment are patients older than 18 years undergoing elective, urgent or emergent surgical interventions for infective endocarditis (IE) or non-infectious valvular heart disease (VHD). The diagnosis of IE will be validated in accordance with the Duke criteria ***[32]***, confirmation of VHD will be accomplished by echocardiography.

The provision of informed consent by the patient or patient’s legal guardian is a pre-requisite for inclusion. Informed consent also comprises patient’s permission for the collection, use and storage of biological material (blood plasma and valve tissue).

Pre-operative examination comprises assessment of surgical risk (EuroScore), pre-surgical co-morbidity (Charlson score) and acute organ dysfunction (SOFA score).

Plasma samples for the determination of baseline levels of inflammatory and vasoactive mediators of interest will be collected within 12 to 24 hours before transfer to the operating theater. Subsequent sampling will occur at the following time points:

- Immediately before connection to the CPB
- 60 minutes after connection to the CPB,
- disconnection of the CPB
- 6, 24 and 48 hours after the end of the operation

The end of the surgical intervention is defined by the time record in the operative report.

The volume of blood samples for the isolation of EDTA plasma needed for determinations of inflammatory and vasoactive mediators will amount to 8 ml at each time point. Blood samples of 5.5 ml volume for pathogen genotyping will be collected 24 hours before surgery, 60 min after connection to the CPB, and 6 and 24 hours after completion of the operation. The total volume of blood collected over the entire study amounts to 78 ml.

Affected valvular tissue for microbiological examination will be collected in the course of surgery.

Post-surgery organ dysfunction will be assessed by SOFA scores taken 24 and 48 hours after the completion of the intervention, and at the time of discharge from the ICU.

Overall duration of CPB will be recorded at the end of the intervention.

Cumulative doses of concomitant medication and the use and duration of renal replacement therapy will be recorded 48 h post-surgery.

| **Flowchart** | **Time point** | | | | | | | | |
| --- | --- | --- | --- | --- | --- | --- | --- | --- | --- |
|  | 24 h before surgery | CPB connection | 60 min on CPB | CPB disconnection | 6 h post-surgery | 24 h post-surgery | 48 h post-surgery | Discharge from ICU | 30 days post-surgery |
| Check inclusion criteria; Informed consent | x |  |  |  |  |  |  |  |  |
| Operative risk assessment (EuroScore) | x |  |  |  |  |  |  |  |  |
| Charlson co-morbidity assessment | x |  |  |  |  |  |  |  |  |
| Organ dysfunction assessment (SOFA) | x |  |  |  |  | x | x | x |  |
| Blood sampling for mediator profiling | x | x | x | x | x | x | x |  |  |
| Blood sampling for pathogen genotyping | x |  | x |  | x | x |  |  |  |
| Tissue sampling for pathogen genotyping |  |  | x |  |  |  |  |  |  |
| Overall duration of CPB |  |  |  | x |  |  |  |  |  |
| Cumulative doses of concomitant medications |  |  |  |  |  |  | x |  |  |
| Use and duration of renal replacement therapy |  |  |  |  |  |  |  | x |  |
| In-hospital mortality |  |  |  |  |  |  |  |  | x |

## Variables

Changes in inflammatory and vasoactive mediators will be monitored at selected intervals during and after the intervention. The resulting temporal profiles are expected to enable the distinction of release episodes related to surgical stress and tissue damage (VHD) from those associated with infection (IE). Besides monitoring of several established markers of inflammation (PCT, CRP, Endothelin, TNFα, IL1β, IL-6, IL-10, IL-18), the study envisages measurements of three vasoactive endogenous compounds (pro-ADM, pro-AVP and pro-ANP) that are affected by both, infection and cardiogenic stressful stimuli. It is also conceivable that, due to their influence on vasomotor regulation, electrolyte and fluid balance, changes in the abundance of the latter compounds may influence constituents of organ dysfunction scores. The integrated expression of mediator release over the entire intervention (i.e. as “area under the curve”) will serve as a proxy of the individual’s humoral responsiveness to the infectious/inflammatory and surgical/CPB challenges.

Day-to-day differences in SOFA scores will be used as indicator of vital organ dysfunction, whereas recording of in-hospital mortality will enable comparisons between predicted and factual disease-associated risk.

The co-variables “Charlson co-morbidity score”, “Cumulative doses of concomitant medications”, “Overall duration of CPB use” and “Use and duration of renal replacement therapy” are of auxiliary importance for the between-patient adjustment of the treatment conditions.

Involved pathogens will be identified by genotyping using Next Generation Sequencing (NGS) of DNA extracted from plasma samples. Analysis will comprise pathogen description, check of congruousness to conventional blood culture data and changes in the pathogen spectrum occurring at selected time points in the course of observation. In addition, the presence and abundance of pathogen-specific transcripts encoding antibiotic resistance factors will be examined in whole blood mRNA extracts.

The abundance of mRNA of inflammatory and vasoactive mediators will be measured in RNA extracts from blood cells and compared with corresponding protein levels measured in plasma samples.

## Data sources

Participants will be recruited based on a confirmed diagnosis in accordance with established criteria and a signed informed consent.

Data capture comprises recording of endpoint parameters at baseline and at defined intervals during and following the surgical intervention, while in ICU. An appraisal of signs of organ dysfunction will also take place at the time of ICU discharge. Information on in-hospital mortality over 30 days will be gathered from patient records or, in case of transfer to other facilities, through confidential inquiries conducted by the Principal Investigator

The influence of potential confounding variables and effect modifiers (e.g. concomitant anticoagulant medication, fluid resuscitation, renal replacement therapy etc.) will be extrapolated from the corresponding specific entries in the eCRF. Co-morbidities that may influence surgical risk and vital organ functions will be deduced from the Charlson scores.

The endpoint indicators have been previously validated and are routinely used. SOFA scoring is a recognized approach to the evaluation of organ dysfunction in critically ill and ICU patients. Blood and tissue processing and storage will occur in accordance with GLP-conform SOPs. Measurements of plasma levels of the inflammatory and vasoactive mediators, pathogen genotyping and abundance of relevant transcripts will be performed in certified laboratories using standardized protocols.

## Study size

A sequential group design will be used, with interim analysis of the parameters of interest being conducted upon data collection from “batches” of 20 patients with IE and VHD, respectively. Comparisons of secretory profiles, represented by the mean “area under the curve” (AUC) for each mediator and patient group, will be performed. Parameters displaying significant between-group difference will be given high priority with regard to subsequent laboratory measurements, in order to accelerate goal achievement and improve study cost allocation. The maximal number of patients to be examined is limited to 60 per group.

## Data management

Individual data will be collected in accordance with the flowchart by means of electronic Case Report Forms (eCRF). For the sake of privacy protection and bias prevention plasma and tissue samples will be transferred to participating laboratories in pseudonymous form (i.e., with numeric labels concealing personal and medical information). Upon receipt of laboratory results in MS Excel format, data transfer into the eCRF will be performed at the Center for Clinical Studies of the Jena University Hospital.

User authentication by password-protected logins and a role-based hierarchical security structure warrants multi-tier prevention of unauthorized data access. Data processing employs the software OpenClinica, which fulfils the regulatory requirements (GCP, 21 CFR Part 11).

Data management will resort to electronic applications. The anonymity of data is warranted in the course of data collection and evaluation (c.f. 10 Protection of Human Subjects). A first plausibility check will take place during raw data input in the eCRF at the investigator site, with missing or implausibly aberrant values signaling imminent correction requirement. Permission for data corrections will be conferred to a restricted number of authorized study personnel (e.g. site investigator, study nurse, data manager). A further verification of plausibility and consistency will be conducted by. A final plausibility check applying a controversy-detection algorithm and the assistance of a medical expert will take place during transfer of external laboratory data and eCRF record consolidation.

## Data analysis

Analysis of profiles of inflammatory and vasoactive mediators will be performed by calculation of the “area under the curve” (AUC) outlined by plasma concentrations measured at consecutive time points. The AUC will be adjusted for baseline values of the mediators and comparisons will be made by the Mann-Whitney U-Test. For the identification of the maximum of inflammatory and vasoactive mediators in each group, measurements at different time points will be pairwise compared by the Wilcoxon signed rank test or paired t-test, if the data are normally distributed.

Differences in mediator levels between the two patient groups are presumed to decrease with time after surgery. At the latest time point of the study (48h post-surgery) baseline-adjusted group differences between mediator levels will be assessed and compared by the Mann-Whitney U-test or the unpaired t-test, as appropriate.

Consecutive changes in SOFA scores (ΔSOFA) at individual post-diagnosis intervals and their mean values calculated for each of two consecutive days will be compared to the scores determined on admission (baseline). Changes in these indices will be examined by means of a linear model with organ dysfunction score baseline adjustment and alternative co-variables (e.g. inflammatory and vasoactive parameters).

Associations between individual post-surgical organ dysfunction scores and the release profiles of selected inflammatory and vasoactive mediators in the course of cardiac surgery will be examined by the Spearman’s rank correlation coefficient.

Data on in-hospital mortality will be presented as percent rates for each study group.

All endpoints will be subjected to descriptive statistical analysis with determination of 95-percent confidence intervals. Between-group comparisons will be preceded by distribution analysis (Shapiro-Wilk Test of normality). Depending on the data distribution, post hoc processing will involve either parametric (t test) or non-parametric (Mann-Whitney U-test) approaches.

Results of pathogen genotyping and gene expression of factors accounting for antibiotic resistance will be denoted as normalized abundance scores; besides individual description, frequency analysis of pathogen occurrence within the study population and principal component analysis for disclosure of relationships between pathogen species and cytokine release profiles will be made, if appropriate.

Correlation between transcript abundance and circulating levels of inflammatory and vasoactive mediators will be examined by calculation of Spearman’s coefficients at corresponding time points.

## Quality control

Quality control measures encompass activities aiming at the preclusion of protocol violations and erroneous data capture and transmission. They include surveillance of

- collection of data within the specified window of time
- accuracy, consistency and plausibility of eCRF records and laboratory data entries
- adherence to regulations on privacy and data protection

Quality control measures will be exerted by the Principal Investigator, the responsible Data Manager and an internal medical expert. Monitoring site visits are not planned for this study.

## Limitations of the research methods

Circulating levels of inflammatory mediators in grave infectious conditions are usually higher than the “intercept points” of most assays; thus, demonstration of subtle differences between single measurements might be impossible. Temporal release profiles of inflammatory biomarkers depend on the duration of the infection and influenced by confounding factors (age, immune status, glucocorticoid therapy etc.). Conceivably, broad inter-individual variations in baseline levels and difficulties in subsequent statistical comparisons should be taken into account.

Pro-ADM, pro-AVP and pro-ANP are degradation-resistant precursors of the proper vasoactive peptides adrenomedullin, vasopressin and atrial natriuretic peptide. Measurements of these precursors serve as surrogates of the biologically active compounds. There is insufficient evidence that precursor levels faithfully reflect the biological efficacy of the vasoactive mediators.

The probable diversity or multiplicity of pathogens may restrict the possibilities for analysis of relationships in samples comprising small number of subjects.

# Protection of human subjects

The study protocol is subject to approval by the IRB of the Friedrich Schiller University, Jena, which will serve as the supervisory ethic institution in charge.

The probability of participation in the study will not influence the physician’s decisions or the patient’s medication.

An informed consent, signed by the patient or his/her legally acceptable representative, also comprising collection and use of biological material and medical data, is a pre-requisite for enrolment. In temporarily disabled patients assent signed by the legally acceptable representative or a signed favorable opinion of a medical consultant will enable the commencement of data collection; the consent of the patient will be sought at the earliest suitable time.

The protection of participant’s privacy will be warranted by ***a)*** pseudonymization of patient personal and medical data and ***b)*** data transmission using secure paradigms. The pseudonymous identity list will assign a four-digit-number to each participant and contain a pre-defined minimum of personal information considered essential for the processing of queries pertaining to accuracy, plausibility and consistency. For communication with the participating laboratories and the Data Management, only the assigned four-digit-numbers will be used. The identity a patient can be disclosed only to the authorized study team members. The identity list and signed consent forms will be stored for at least 10 years in a mode that precludes access of non-authorized persons.

The owner of the study data is the Principal Investigator. Documentation will take place at the Center for Clinical Studies of the Jena University Hospital. Study data will be processed automatically, with scheduled regular backups, and made accessible to a limited number of staff members, who are directly involved in this project. Data media are stored in a secured space on the premises of the Center for Clinical Studies, with access permitted only to the system administrator.

Publication of study data will occur in aggregated form, without disclosure of details of subjects and participating investigation sites. Case reports will be published under the condition of anonymity. For the dissemination of study data to external requestors, approval of the IRB will be solicited.

The regulations pertaining to patients’ welfare and privacy protection are in compliance with the international and national norms and legislation (Helsinki Declaration, ICH Guideline for GCP).

# Management and reporting of adverse events/adverse reactions

In view of the study design, as well as the complex pathology and the multitude of simultaneously used therapeutic agents, no registration of treatment-emergent AEs is planned.

# Plans for disseminating and communicating study results

Study results will be disseminated in aggregated form as scientific publications or as anonymous case reports.

External interested parties may receive on demand and on condition of approval by the IRB excerpts from study data pertaining to a previously defined topic.

Representatives of the IRB or the supervisory authorities are entitled to study data insight in the frame of official audits.

# References

| 1 | Prendergast BD and Tornos P | *Circulation 121: 1141-1152 2010* |
| --- | --- | --- |
| 2 | Tleyjeh IM et al. | *Chest 132: 1025-1035 2007* |
| 3 | Yew HS and Murdoch DR | *Curr Inf Dis Rep 14: 367-372 2012* |
| 4 | Murdoch DR et al. | *Arch Intern Med 169: 463-473 2009* |
| 5 | Galvez-Acebal J et al. | *BMC Infect Dis 10: 17 2010* |
| 6 | Gelsomino S et al. | *Ann Thorac Surg 93: 1469-1476 2012* |
| 7 | Musci M et al. | *Clin Res Cardiol 98: 443-450 2009* |
| 8 | Mourvillier B et al. | *Intensive Care Med 30: 2046-2052 2004* |
| 9 | Werdan K et al. | *Nat Rev Cardiol 11: 35-50 2014* |
| 10 | Diab M et al. | *Eur J Cardiothorac Surg 44: e289-e294 2013* |
| 11 | Boyle EM et al. | *Ann Thorac Surg 63: 277-284 1997* |
| 12 | Alter P et al. | *Am J Cardiol 89: 1400-1404 2002* |
| 13 | Hotchkiss RS and Karl IE | *N Engl J Med 348: 138-150 2003* |
| 14 | Tamayo E et al. | *Eur Cytokine Netw 22: 82-87 2011* |
| 15 | Bustamante J et al. | *APMIS 122: 526-529* |
| 16 | Ekdahl c et al. | *Scand J Infect Dis 34: 759-762* |
| 17 | Tisoncik JR et al. | *Microbiol Mol Biol Rev 76: 16-32 2012* |
| 18 | Cohen J | *Nature 420: 885-891 2002* |
| 19 | Born F et al. | *Kardiotechnik 2: 41-46 2014* |
| 20 | Mohamed M et al. | *Cytokine 39: 171-177 2007* |
| 21 | De Dooy J et al. | *Pediatr Res 56: 547-552 2004* |
| 22 | Tattevin P et al. | *Med Mal Infect dx.doi.org/10.1016/j.medmal.2014.11.003* |
| 23 | McLean D et al. | *Nat Rev Microbiology 7: 287-296 2009* |
| 24 | Christ-Crain M et al. | *Crit Care 9: R816-R824 2005* |
| 25 | Guignant C et al. | *Intensive Care Med 35: 1859-1867 2009* |
| 26 | Suberviola B et al. | *Swiss Med Wkly 142: w13542 2012* |
| 27 | Moura LM et al. | *Exp Rev Cardiovasc Ther 6: 945-954 2008* |
| 28 | Tang F et al. | *Horm Metab Res 37: 585-588 2005* |
| 29 | Bhalla V et al. | *Crit Care Med 32: 1787-1789 2004* |
| 30 | Katan M and Christ-Crain M | *Swiss Med Wkly 140: w13101 2010* |
| 31 | Nicholls MG et al. | *Hypertens Res 26 (Suppl) S135-S140 2003* |
| 32 | Li JS et al. | *Clin Infect Dis 30: 633-638 2000* |
