# Supplementary material for: Changes in inflammatory and vasoactive mediator profiles during valvular surgery with or without infective endocarditis: A case control pilot study
Source: PLoS One. 2020 Feb 3;15(2):e0228286. doi: 10.1371/journal.pone.0228286 (PMC6996967; doi:10.1371/journal.pone.0228286)
Supplement: S1 Table — (RTF) [file pone.0228286.s014.rtf]

S1 Table: Pre-operative characteristics of patients with infective endocarditis
	n	%	
Mode of Acquisition				
	nosocomial	2	10.0	
	non nosocomial	10	50.0	
	Community  acquired	8	40.0	
	drug abuse	0	0.0	
Indication for operation				
	heart failure	11	55.0	
	uncontrolled infection	7	35.0	
	prevention of embolism	12	60.0	
Symptoms				
	Fever	8	40.0	
	Nausea	9	45.0	
	Deterioration of general condition	20	100.0	
	Neurological events	6	30.0	
	B-symptoms	2	10.0	
	Dyspnea	15	75.0	
Prosthetic endocaridtis		6	30.0	
vegetation		20	100.0	
abscess		6	30.0	


Euroscore II: Alter

Age (Years)	Endocarditis	VHD	
MEAN	63.65	66.55	
STDDEV	9.522	10.303	
N	20	20	
