# Supplementary material for: Changes in inflammatory and vasoactive mediator profiles during valvular surgery with or without infective endocarditis: A case control pilot study
Source: PLoS One. 2020 Feb 3;15(2):e0228286. doi: 10.1371/journal.pone.0228286 (PMC6996967; doi:10.1371/journal.pone.0228286)
Supplement: S2 Table — (DOCX) [file pone.0228286.s015.docx]

S2 Table. Spearman correlation analysis between the maximal level of inflammatory markers during CPB and cumulative doses of vasopressor or catecholamine doses at 6 and 24h postoperatively

|  |  | Norepinephrine | | Epinephrine | | Vasopressin | |
| --- | --- | --- | --- | --- | --- | --- | --- |
|  |  | 6h | 24h | 6h | 24h | 6h | 24h |
| MRproANP [pmol/l] | rs  p | **0.59487**  **<.0001** | **0.60226**  **<.0001** | 0.34150  0.0310 | 0.33863  0.0350 | 0.26903  0.0932 | 0.34807  0.0299 |
| MRproADM [nmol/l] | rs  p | **0.71936**  **<0.0001** | **0.74336**  **<0.0001** | 0.49339  0.0012 | 0.46832  0.0026 | 0.43000  0.0056 | 0.43291  0.0059 |
| CTproET1 [pmol/l] | rs  p | 0.35921  0.0228 | 0.47087  0.0025 | 0.36049  0.0223 | **0.53166**  **0.0005** | 0.14039  0.3876 | 0.22055  0.1773 |
| PCTsen [μg/l] | rs  p | 0.49927  0.0010 | 0.66805  <0.0001 | 0.33380  0.0353 | 0.41719  0.0082 | 0.35947  0.0227 | 0.31880  0.0479 |
| CRPus [μg/ml] | rs  p | 0.29551  0.0641 | -0.12318  0.4550 | 0.18217  0.2606 | 0.06389  0.6992 | 0.18872  0.2435 | -0.09285  0.5740 |
| proAVP [pmol/l] | rs  p | -0.08284  0.6113 | 0.36146  0.0238 | -0.09442  0.5622 | 0.20811  0.2036 | -0.20863  0.1964 | 0.00540  0.9740 |
| IL-1 β [pg/mL] | rs  p | 0.01198  0.9415 | -0.06726  0.6841 | 0.03228  0.8433 | 0.15883  0.3342 | 0.11820  0.4676 | -0.14620  0.3745 |
| IL-6 [pg/mL] | rs  p | 0.39542  0.0116 | 0.29759  0.0658 | 0.26632  0.0967 | 0.04593  0.7813 | 0.22463  0.1635 | 0.07596  0.6458 |
| IL-10 [pg/mL] | rs  p | 0.48991  0.0013 | 0.51613  0.0008 | 0.19504  0.2278 | 0.42484  0.0070 | 0.23840  0.1385 | 0.46048  0.0032 |
| TNF-α [pg/mL] | Rs  p | 0.42349  0.0065 | 0.37867  0.0174 | 0.23129  0.1510 | 0.26849  0.0984 | 0.37045  0.0186 | 0.22317  0.1720 |
| IL-18 [pg/mL] | rs  p | 0.34655  0.0285 | 0.37532  0.0186 | 0.20167  0.2121 | 0.25048  0.1241 | 0.21059  0.1921 | 0.21271  0.1936 |

**This is the Table legend.**

CPB: cardiopulmonary bypass; rs: Spearman´s Rank Correlation Coefficient;IL: inteleukin; MR-proANP: midregional pro adrenomedullin; MR-proANP: midregional pro atrial natriuretic peptide; CT-proAVP: copeptin midregional pro vasopressin; CT-proET1: C-terminal pro endothelin; TNF: tumor necrosis factor
