# Supplementary material for: Changes in inflammatory and vasoactive mediator profiles during valvular surgery with or without infective endocarditis: A case control pilot study
Source: PLoS One. 2020 Feb 3;15(2):e0228286. doi: 10.1371/journal.pone.0228286 (PMC6996967; doi:10.1371/journal.pone.0228286)
Supplement: S3 Table — (DOCX) [file pone.0228286.s016.docx]

S3 Table. Spearman correlation analysis between the time from diagnosis of infective endocarditis to operation and the levels of cytokines and vasoactive peptides at defined time points

|  |  | 24h Pre-op | Start CPB | 60 minutes CPB | End of CPB | 6h postop | 24h postop | 48h postop |
| --- | --- | --- | --- | --- | --- | --- | --- | --- |
| MR-proANP | rs | 0.105 | 0.089 | 0.093 | 0.184 | 0.048 | -0.066 | -0.055 |
|  | p | 0.659 | 0.708 | 0.732 | 0.450 | 0.841 | 0.789 | 0.827 |
| MR-proADM | rs | -0.084 | 0.005 | 0.107 | 0.038 | -0.011 | -0.089 | -0.089 |
|  | p | 0.725 | 0.982 | 0.695 | 0.876 | 0.962 | 0.716 | 0.726 |
| CT-proET-1 | rs | -0.0632 | 0.103 | -0.089 | 0.063 | -0.062 | -0.113 | -0.019 |
|  | p | 0.791 | 0.711 | 0.740 | 0.797 | 0.796 | 0.645 | 0.9376 |
| PCT | rs | -0.120 | -0.055 | -0.190 | -0.293 | 0.044 | 0.060 | 0.134 |
|  | p | 0.615 | 0.818 | 0.480 | 0.223 | 0.853 | 0.808 | 0.597 |
| CRP | rs | 0.091 | 0.089 | 0.035 | 0.212 | 0.151 | -0.272 | -0.237 |
|  | p | 0.704 | 0.711 | 0.895 | 0.383 | 0.526 | 0.260 | 0.343 |
| CT-proAVP | rs | -0.223 | -0.098 | -0.025 | -0.054 | 0.083 | -0.170 | -0.091 |
|  | p | 0.344 | 0.680 | 0.925 | 0.825 | 0.7278 | 0.486 | 0.7196 |
| **IL1** | rs | -0.468 | -0.470 | **-0.536** | -0.364 | -0.368 | -0.412 | -0.388 |
|  | p | 0.037 | 0.036 | 0.0324 | 0.242 | 0.111 | 0.080 | 0.111 |
| IL-6 | rs | -0.328 | -0.293 | -0.439 | -0.282 | -0.127 | -0.127 | 0.046 |
|  | p | 0.158 | 0.209 | 0.088 | 0.242 | 0.593 | 0.603 | 0.856 |
| IL-10 | rs | -0.474 | -0.274 | -0.239 | -0.256 | 0.153 | -0.374 | -0.419 |
|  | p | 0.035 | 0.242 | 0.372 | 0.125 | 0.519 | 0.115 | 0.083 |
| **TNF-α** | rs | -0.010 | -0.079 | -0.215 | -0.414 | -0.124 | **-0.550** | -0.27091 |
|  | p | 0.968 | 0.742 | 0.4236 | 0.078 | 0.603 | 0.0147 | 0.2769 |
| **IL18** | rs | -0.344 | -0.357 | 0.047 | -0.255 | -0.351 | **-0.598** | **-0.591** |
|  | p | 0.138 | 0.122 | 0.859 | 0.291 | 0.130 | 0.007 | 0.010 |

**This is the Table legend.** CPB: cardiopulmonary bypass; rs: Spearman´s Rank Correlation Coefficient;IL: inteleukin; MR-proANP: midregional pro adrenomedullin; MR-proANP: midregional pro atrial natriuretic peptide; CT-proAVP: copeptin midregional pro vasopressin; CT-proET1: C-terminal pro endothelin; TNF: tumor necrosis factor
