# Supplementary material for: Changes in inflammatory and vasoactive mediator profiles during valvular surgery with or without infective endocarditis: A case control pilot study
Source: PLoS One. 2020 Feb 3;15(2):e0228286. doi: 10.1371/journal.pone.0228286 (PMC6996967; doi:10.1371/journal.pone.0228286)
Supplement: S4 Table — (DOCX) [file pone.0228286.s017.docx]

S4 Table. Spearman correlation analysis between the duration of antibiotic therapy and the levels of cytokines and vasoactive peptides

|  |  | **24h Pre-OP** | **Start CPB** | **60 minutes CPB** | **End of CPB** | **6h post-OP** | **24h post-OP** | **48h post-OP** |
| --- | --- | --- | --- | --- | --- | --- | --- | --- |
| **MRproANP** | **r_s_** | 0.372 | 0.154 | 0.114 | 0.088 | -0.145 | -0.231 | -0.188 |
|  | **p** | 0.128 | 0.543 | 0.674 | 0.738 | 0.565 | 0.373 | 0.486 |
| **MRproADM** | **r_s_** | -0.029 | -0.122 | -0.127 | -0.049 | -0.141 | -0.153 | -0.176 |
|  | **p** | 0.909 | 0.629 | 0.638 | 0.851 | 0.577 | 0.558 | 0.515 |
| **CTproET1** | **r_s_** | 0.096 | -0.025 | 0.009 | 0.112 | -0.119 | -0.149 | -0.049 |
|  | **p** | 0.703 | 0.922 | 0.974 | 0.668 | 0.637 | 0.568 | 0.858 |
| **PCTsen** | **r_s_** | -0.157 | -0.113 | -0.096 | -0.376 | 0.020 | 0.127 | 0.242 |
|  | **p** | 0.535 | 0.655 | 0.723 | 0.137 | 0.938 | 0.627 | 0.366 |
| **CRPus** | **r_s_** | -0.090 | -0.228 | -0.101 | -0.312 | 0.039 | -0.245 | -0.170 |
|  | **p** | 0.722 | 0.362 | 0.711 | 0.223 | 0.877 | 0.343 | 0.529 |
| **proAVP** | **r_s_** | -0.174 | 0.009 | 0.169 | -0.125 | 0.218 | -0.109 | 0.028 |
|  | **p** | 0.489 | 0.971 | 0.532 | 0.634 | 0.385 | 0.679 | 0.918 |
| **IL1** | **r_s_** | 0.258 | -0.362 | -0.103 | -0.221 | -0.138 | -0.234 | -0.114 |
|  | **p** | 0.300 | 0.140 | 0.705 | 0.395 | 0.586 | 0.366 | 0.673 |
| **IL6** | **r_s_** | -0.362 | -0.215 | -0.022 | -0.287 | -0.329 | -0.116 | 0.353 |
|  | **p** | 0.140 | 0.391 | 0.935 | 0.264 | 0.183 | 0.658 | 0.180 |
| **IL10** | **r_s_** | -0.124 | -0.023 | 0.230 | -0.150 | 0.201 | -0.185 | -0.329 |
|  | **p** | 0.624 | 0.928 | 0.391 | 0.564 | 0.423 | 0.478 | 0.214 |
| **TNFalpha** | **r_s_** | 0.150 | -0.051 | 0.117 | 0.003 | -0.162 | -0.222 | 0.062 |
|  | **p** | 0.552 | 0.842 | 0.666 | 0.990 | 0.520 | 0.392 | 0.821 |
| **IL18** | **r_s_** | -0.338 | -0.320 | -0.123 | -0.265 | -0.349 | -0.455 | -0.470 |
|  | **p** | 0.170 | 0.196 | 0.650 | 0.304 | 0.156 | 0.067 | 0.066 |

CPB: cardiopulmonary bypass; rs: Spearman´s Rank Correlation Coefficient;IL: inteleukin; MR-proANP: midregional pro adrenomedullin; MR-proANP: midregional pro atrial natriuretic peptide; CT-proAVP: copeptin midregional pro vasopressin; CT-proET1: C-terminal pro endothelin; TNF: tumor necrosis factor
