# Supplementary material for: Changes in inflammatory and vasoactive mediator profiles during valvular surgery with or without infective endocarditis: A case control pilot study
Source: PLoS One. 2020 Feb 3;15(2):e0228286. doi: 10.1371/journal.pone.0228286 (PMC6996967; doi:10.1371/journal.pone.0228286)
Supplement: S5 Table — (DOCX) [file pone.0228286.s018.docx]

S5 Table. Comparison of cytokines and vasoactive peptides between patients operated in minimally invasive technique compared to those operated in sternotomy within the VHD group.

|  | | **Not minimal invasive** | | **Minimal invasive** | |  |
| --- | --- | --- | --- | --- | --- | --- |
| **Marker** | **Time points of comparison** | **Median** | **IQR** | **Median** | **IQR** | **U test, p-value** |
| CRPus | preOP 24h | 7.56 | 9.45 | 1.92 | 2.96 | 0.184 |
|  | CPB connection | 5.15 | 6.34 | 1.19 | 4.07 | 0.138 |
|  | 60min on CPB | 4.31 | 4.42 | 0.92 | 2.73 | 0.087 |
|  | CPB disconnection | 4.06 | 5.19 | 0.96 | 2.99 | 0.119 |
|  | 6h post surgery | 6.61 | 16.22 | 6.90 | 8.43 | 0.909 |
|  | 24h post surgery | 103.22 | 40.27 | 117.51 | 61.99 | 0.342 |
|  | 48h post surgery | 185.72 | 68.51 | 209.37 | 59.91 | 0.621 |
| CTproET1 | preOP 24h | 90.50 | 90.44 | 84.32 | 32.36 | 0.790 |
|  | CPB connection | 76.46 | 37.62 | 60.28 | 34.12 | 0.676 |
|  | 60min on CPB | 70.30 | 37.63 | 70.93 | 30.97 | 0.790 |
|  | CPB disconnection | 68.96 | 35.07 | 69.07 | 42.24 | 0.732 |
|  | 6h post surgery | 142.36 | 51.25 | 144.77 | 68.83 | 0.676 |
|  | 24h post surgery | 130.84 | 58.33 | 125.39 | 49.52 | 0.849 |
|  | 48h post surgery | 115.51 | 125.46 | 115.89 | 44.31 | 0.909 |
| IL1 | preOP 24h | 2.00 | 0.00 | 2.00 | 0.00 | 1.000 |
|  | CPB connection | 2.00 | 0.00 | 2.00 | 0.00 | 0.827 |
|  | 60min on CPB | 2.00 | 0.00 | 2.00 | 0.00 | 1.000 |
|  | CPB disconnection | 2.00 | 0.00 | 2.00 | 0.00 | 1.000 |
|  | 6h post surgery | 2.00 | 0.00 | 2.00 | 0.00 | 0.269 |
|  | 24h post surgery | 2.00 | 0.00 | 2.00 | 0.00 | 1.000 |
|  | 48h post surgery | 2.00 | 0.00 | 2.00 | 0.00 | 1.000 |
| IL10 | preOP 24h | 2.20 | 0.00 | 2.20 | 0.00 | 1.000 |
|  | CPB connection | 2.20 | 0.00 | 2.20 | 0.00 | 1.000 |
|  | 60min on CPB | 8.81 | 12.63 | 11.41 | 19.80 | 0.732 |
|  | CPB disconnection | 20.08 | 37.74 | 43.90 | 79.08 | 0.470 |
|  | 6h post surgery | 30.66 | 64.22 | 2.95 | 13.33 | 0.183 |
|  | 24h post surgery | 3.70 | 6.32 | 2.20 | 0.27 | **0.031** |
|  | 48h post surgery | 2.20 | 0.89 | 2.20 | 0.00 | 0.142 |
| IL18 | preOP 24h | 66.95 | 40.74 | 47.07 | 47.43 | 0.790 |
|  | CPB connection | 66.17 | 45.95 | 65.49 | 50.51 | 0.676 |
|  | 60min on CPB | 54.97 | 32.78 | 44.54 | 49.45 | 0.790 |
|  | CPB disconnection | 57.69 | 48.38 | 47.99 | 45.67 | 0.761 |
|  | 6h post surgery | 90.96 | 97.18 | 68.10 | 59.72 | 0.518 |
|  | 24h post surgery | 146.81 | 126.38 | 87.04 | 111.01 | 0.425 |
|  | 48h post surgery | 126.08 | 139.39 | 58.60 | 69.76 | 0.184 |
| IL6 | preOP 24h | 9.20 | 0.00 | 9.20 | 0.00 | 1.000 |
|  | CPB connection | 9.20 | 0.00 | 9.20 | 0.00 | 0.109 |
|  | 60min on CPB | 13.40 | 8.31 | 9.20 | 6.54 | 0.383 |
|  | CPB disconnection | 41.53 | 55.48 | 19.25 | 28.65 | 0.270 |
|  | 6h post surgery | 204.30 | 82.07 | 110.30 | 156.14 | 0.074 |
|  | 24h post surgery | 196.96 | 99.13 | 101.61 | 155.27 | 0.087 |
|  | 48h post surgery | 84.84 | 49.79 | 60.23 | 42.52 | 0.342 |
| MRproADM | preOP 24h | 1.20 | 0.52 | 0.93 | 0.26 | 0.470 |
|  | CPB connection | 0.86 | 0.20 | 0.79 | 0.31 | 0.909 |
|  | 60min on CPB | 0.88 | 0.16 | 0.98 | 0.29 | 0.518 |
|  | CPB disconnection | 1.03 | 0.27 | 1.13 | 0.66 | 0.382 |
|  | 6h post surgery | 1.82 | 1.61 | 1.38 | 2.05 | 0.569 |
|  | 24h post surgery | 2.57 | 2.46 | 1.46 | 0.50 | 0.342 |
|  | 48h post surgery | 2.49 | 2.95 | 1.62 | 0.68 | 0.470 |
| MRproANP | preOP 24h | 213.06 | 257.14 | 119.80 | 116.41 | 0.425 |
|  | CPB connection | 199.23 | 255.85 | 126.87 | 137.06 | 0.342 |
|  | 60min on CPB | 171.89 | 172.36 | 128.15 | 285.82 | 0.382 |
|  | CPB disconnection | 332.99 | 325.06 | 263.25 | 291.45 | 0.849 |
|  | 6h post surgery | 237.61 | 313.43 | 150.75 | 72.36 | 0.849 |
|  | 24h post surgery | 227.68 | 322.31 | 193.51 | 75.66 | 0.849 |
|  | 48h post surgery | 287.16 | 252.50 | 180.43 | 145.92 | 0.470 |
| PCTsen | preOP 24h | 0.07 | 0.07 | 0.09 | 0.08 | 0.790 |
|  | CPB connection | 0.07 | 0.05 | 0.08 | 0.05 | 0.470 |
|  | 60min on CPB | 0.04 | 0.06 | 0.08 | 0.07 | 0.063 |
|  | CPB disconnection | 0.06 | 0.03 | 0.09 | 0.07 | 0.518 |
|  | 6h post surgery | 0.26 | 0.79 | 0.58 | 1.71 | 0.160 |
|  | 24h post surgery | 2.52 | 9.06 | 1.32 | 2.46 | 0.569 |
|  | 48h post surgery | 1.89 | 6.21 | 0.86 | 1.17 | 0.425 |
| TNFalpha | preOP 24h | 7.20 | 0.00 | 7.20 | 0.00 | 1.000 |
|  | CPB connection | 7.20 | 0.00 | 7.20 | 0.00 | 0.269 |
|  | 60min on CPB | 7.20 | 0.00 | 7.20 | 0.00 | 1.000 |
|  | CPB disconnection | 7.20 | 0.00 | 7.20 | 0.00 | 1.000 |
|  | 6h post surgery | 7.20 | 0.00 | 7.20 | 0.00 | 0.269 |
|  | 24h post surgery | 7.20 | 0.00 | 7.20 | 0.00 | 0.582 |
|  | 48h post surgery | 7.20 | 0.00 | 7.20 | 0.00 | 1.000 |
| proAVP | preOP 24h | 9.23 | 13.57 | 10.00 | 13.98 | 0.470 |
|  | CPB connection | 35.72 | 109.18 | 42.15 | 105.16 | 0.909 |
|  | 60min on CPB | 115.28 | 80.07 | 70.77 | 228.18 | 0.342 |
|  | CPB disconnection | 68.42 | 64.54 | 77.88 | 175.63 | 0.970 |
|  | 6h post surgery | 295.41 | 321.99 | 309.84 | 405.83 | 0.790 |
|  | 24h post surgery | 105.90 | 109.80 | 50.09 | 68.14 | 0.342 |
|  | 48h post surgery | 46.26 | 116.31 | 19.79 | 30.60 | 0.102 |
